# FASE-family and social engagement model for prevention and management of self harm behavior–a study protocol for cluster randomized control trial in India

**DOI:** 10.3389/fpsyt.2022.915568

**Published:** 2022-07-28

**Authors:** Saju Madavanakadu Devassy, Lorane Scaria, Anuja Maria Benny, Natania Cheguvera, Jaicob Varghese, Lynette Joubert

**Affiliations:** ^1^Department of Social Work, Rajagiri College of Social Sciences (Autonomous), Kochi, India; ^2^Department of Social Work, Melbourne School of Health Sciences at the University of Melbourne, Carlton, VIC, Australia; ^3^International Centre for Consortium Research in Social Care (ICRS), Rajagiri College of Social Sciences (Autonomous), Kochi, India; ^4^Department of Critical Care Medicine, Rajagiri Hospital, Kochi, India

**Keywords:** suicide prevention, suicide behavior management, family-based intervention, social support, emergency care, India, public mental health

## Abstract

**Background:**

Suicide is a substantial public health concern for countries worldwide. Effective preventive and curative interventions for self-harm behavior (SHB) are imperative for nations with an alarmingly high rate of suicide and self-harm behaviors. The intervention protocol named *FASE (Family and Social Engagement)* consists of comprehensive assessment, Attachment-Based Family Therapy (ABFT), and community linkages for people presenting with suicide or self-harm in emergency departments of tertiary hospitals.

**Methods:**

This article reports the design and protocol for a cluster randomized control trial for suicide prevention and management. After the developed intervention is pilot tested in a tertiary hospital in Kerala, the intervention will be scaled up to be implemented in various tertiary hospitals in Kerala. Each hospital emergency department will be considered a cluster, and these clusters will be randomized to the intervention group and control group in a 1:1 ratio. The eligible people from the intervention clusters will undergo a baseline assessment, a structured moderate intense intervention with twelve sessions spread across 6 months by the trained social workers supervised by the Mental health team, and a follow-up assessment at the end. Participants will be recruited after obtaining consent and explaining the study. The primary outcome includes suicidality measured by the Depressive Symptom Inventory–Suicidality Subscale (DSI-SS), Depression, Anxiety and Stress Scale (DASS), MOS Social Support Survey, and Brief resilience scale (BRS).

**Discussion:**

Knowledge generated from this trial can significantly affect new programmatic policy and clinical guidelines that will improve the reduction of suicide rates in the country.

**Trial registration:**

Prospectively registered in Clinical Trial Registry India (ICMR-NIMS) on 18/10/2021 (ref number- REF/2021/10/048264).

## Introduction

Suicide is a global and significant public health concern worldwide, and its prevention has become a priority on the international public health agenda. Moreover, an aspirational Sustainable Development Goal (SDG) of a one-third reduction in the suicide death rate (SDR) by 2030 (1) compels the member nations to initiate proactive steps to achieve the global targets. The preventive measures are more important in Southeast Asia, accounting for roughly 40% of the estimated 800 000 annual suicide deaths globally. India has the highest suicide rate in South East Asia (2). Furthermore, India has the third-highest female suicide rate of 14.7%, and 71% of them are done by individuals below 44 years (2) imposes a substantial social, economic, and political burden on society. There are indications that for each adult who died by suicide, more than 20 others may have been attempting suicide (3). For each suicide, approximately 135 people are affected with intense grief or other emotional problems (4). Despite these galvanizing rates, India's suicide preventive intervention programs are not promising due to slow coordination at the national and state levels (5, 6). However, the recent decriminalization of suicide in India's Mental Healthcare Act (7) is a proactive step, making the government duty-bound to provide care, treatment, and rehabilitation, instead of penalizing the individual who has attempted suicide.

Alarmingly high suicide rates, global commitments, and paradigm shifts in government policies in India call for an urgent yet well-coordinated suicide prevention and management model to consider the issue of suicide a priority condition. Though the study of suicide and suicidal behavior has been approached from various theoretical models, including cognitive (8, 9) and dialectical behavior (10, 11) models, they often have little clinical efficacy or direct application in the Indian context. There is considerable evidence of the significance of family involvement (12) and increased family support (13) in reducing the risk and facilitating the management of suicidal thoughts and behaviors in individuals. The FASE model is designed as a family-based intervention due to the collectivist mindset of the Indian population. Attachment-based family therapy (ABFT) is a psychological component of the intervention to rebuild meaningful relationships and resolve family ruptures (14). Further, the social and economic issues are dealt with by utilizing the untapped family resources and customizing the pathways or linkages to community resources (15). Therefore, the proposed model consists of an indicated suicide prevention model comprising of patient engagement, ABFT, and formal and informal family and community resource linkages with 1 to 6 months of low-intensity monitoring and follow-up.

The comprehensive mental health assessment and active post-hospital psychosocial care by appropriately trained personnel are the primary prevention strategy to reduce future suicidal ideation and self-harm (16). As a secondary prevention strategy, the project clinicians will provide ABFT with a specific focus on strengthening family involvement. Community linkage with formal services such as government and employment; informal services like non-government and voluntary organizations; community health services, social support service agencies, and financial aid will act as the tertiary and rehabilitative strategy within the model. Thus, this intervention can provide various levels of prevention to people with self-harm behaviors. None of these elements has previously been combined to intervene for suicidal or self-harm behaviors in the Indian population.

This study will be conducted among an identified at-risk and vulnerable group of people presented at emergency. Existing evidence shows that the effective management of self-harm Behavior (SHB) to reduce the risk of subsequent suicide must be an essential element of suicide prevention strategies since self-harm is the most potent risk factor for future suicide (17).

## Materials and methods

### Aim of the study

The research has two aims, to build evidence on the effectiveness of an Attachment-Based Family Therapy and Resource linkage model to prevent suicide in people presenting with self-harm behaviors in emergency departments at tertiary hospitals in Kerala; Secondly, to evaluate the efficacy of the process of delivery of services and assess the cost-effectiveness of sharing the care task with the social work trainees supervised by the mental health team, through a continuum of care from critical health care to family-based services. The research team has been developing this model for over the past 2 years. The results of this feasibility trial would provide insights into the model's effectiveness in responding to multiple and complex psychological and social precipitants to deliberate self-harm that are currently ignored in general health services.

### Objectives of the study

The major objectives of the study include

- Evaluate the contribution of psychosocial factors to self-harm behavior and the role of psychosocial aspects in preventing suicide.
- Develop the ABFT and Community Linkage model for the prevention of suicide in a group of people presenting with self-harm behavior at emergency departments;
- Compare the treatment (TAU) as the usual plus FASE intervention with the TAU using a cluster RCT design framework;
- Develop the systems and processes appropriate to deliver the services for people presenting with self-harm behavior at emergency to integrate a psychosocial intervention that would have broad relevance and application nationally and in low and middle-income countries;
- Assess the proposed model's cost-effectiveness for managing self-harm behavior focused on a seamless continuum of care from acute health to community services.

### Trial design

The study involves three phases;

- Phase 1: development of a family-based intervention with the three components, Comprehensive psychosocial assessment and assertive engagement, Attachment-Based Family Therapy (ABFT), and community resource linkages.
- Phase 2: the feasibility trial of the developed intervention, and
- Phase 3: the cluster randomized control trial of suicide prevention intervention among randomly selected tertiary hospitals in Kerala.

In phase one, the development of intervention components includes a need assessment survey followed by a rapid systematic review of the preventive interventions for suicide and self-harm. A qualitative and quantitative need assessment was conducted among 44 patients who presented with self-harm behaviors in a tertiary hospital in Kerala between October and December 2020 (results submitted elsewhere). The rapid systematic review and the assessment findings guided the components of the intervention. In the second phase, the developed intervention will be tested for feasibility and effectiveness in a tertiary hospital in Kerala using a pre-post interventional design. Phase three comprises the experimental trial of the finalized care package using a cluster-randomized trial in different tertiary hospitals in Kerala.

### Study setting

**Figure 1:**
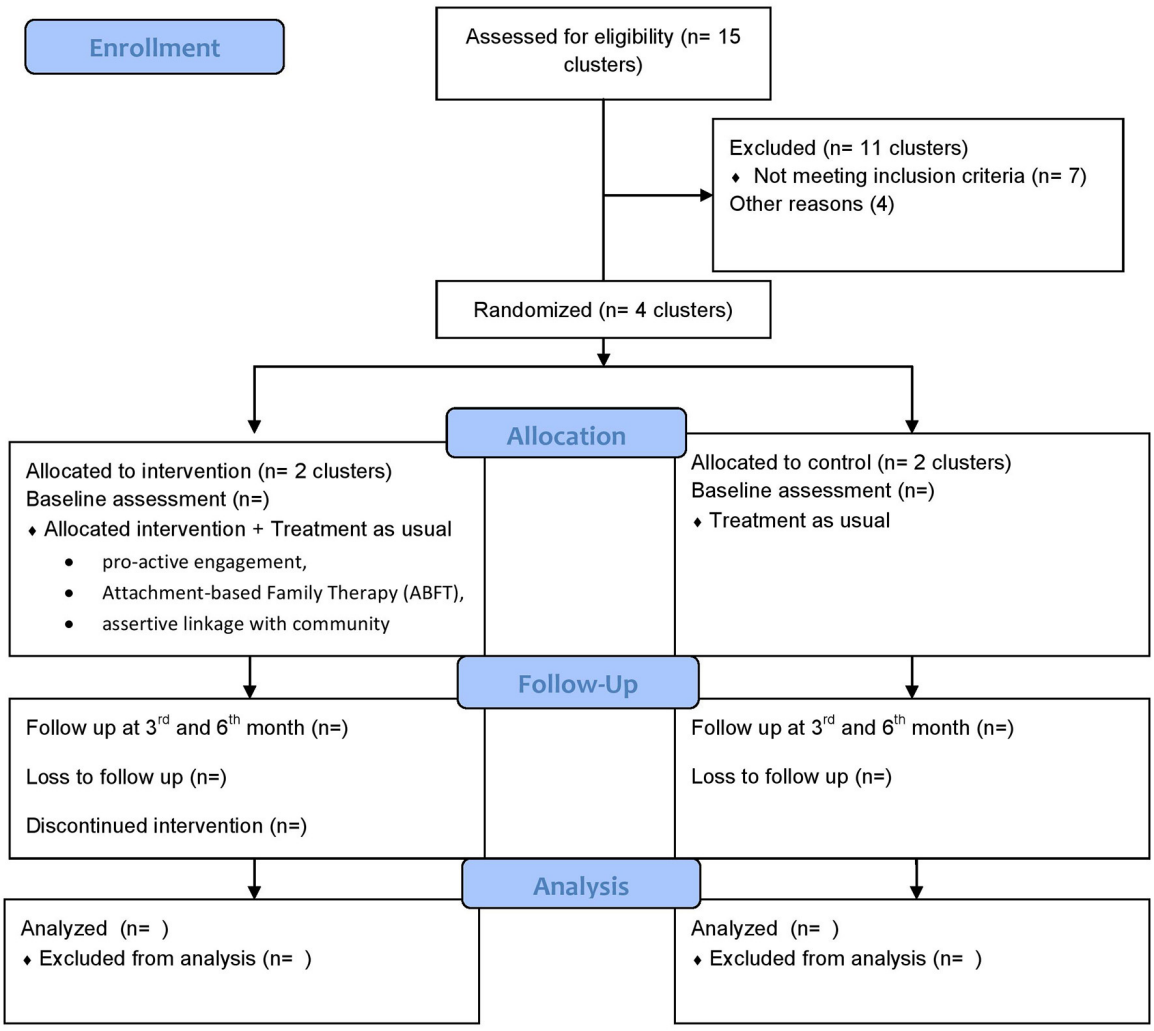
Consort flow diagram.

A list of NABH (National Accreditation Board for Hospitals and Healthcare) Accredited hospitals from Kerala state, India, will be prepared to select the intervention clusters. The inclusion criteria for selecting hospitals will be any NABH accredited Tertiary referral multi-speciality hospital with a minimum of 500 inpatient beds. Eligible hospitals will be randomized to intervention or control groups in a 1:1 allocation ratio. We will sign a referral agreement with the emergency department in charge and their team after explaining to them the purpose. Emergency departments of the chosen hospitals will be considered as individual clusters, and all those who presented to the emergency departments of these hospitals after serious suicidal attempts will be assessed by the professional social workers working in the department. The Social Workers will induct the people and their family members after educating them about the purpose of the study and getting informed consent. The consented people from the experimental cluster will receive the treatment as usual (TAU) plus FASE intervention, and the people belongs the control clusters will receive the treatment as usual (TAU) interventions. The stages of enrolment, intervention, and assessment can be seen in Figure 1.

### Participant recruitment

A feasibility trial will be performed in one of the clusters among patients presenting with self-harm behaviors at their emergency department. Social workers under the supervision of the head of the department of critical care and emergencies in the hospital and the project lead will form a multidisciplinary team to run the project. Professional social workers employed in the hospital are responsible for recruitment, baseline and follow-up assessments, and initial interventions. The mental health team will confirm the caseness of each potential participant after scrutiny of the filled-up assessment framework. The disagreements, if any, will be sorted out by the confirmatory assessment by the mental health team. As part of their field placement, a pool of social work trainees from the social work institutions across the State will deliver the follow-up telephonic interventions. Baseline and 6-month follow-up assessments will be performed using a set of standardized questionnaires. After completing the 6-month-long feasibility trial, the intervention will be tested in the emergency departments of randomly selected clusters.

Criteria for the participants of the cluster-randomized trial include:

The participants should fulfill the standard definition of suicide attempt: “An act with a non-fatal outcome, in which an individual deliberately initiates a non-habitual behavior that without intervention from others will cause self-harm, or deliberately ingests a substance above the prescribed or generally recognized therapeutic dosage, and which is aimed at realizing changes which the subject desired *via* the actual or expected physical consequences” (18).

Inclusion criteria is as follows:

- Patients presenting with attempted suicide or self-harm behaviors at emergency departments of the selected hospitals will be included.
- We will include patients with age over 18 with non-habitual deliberate self-harm, and serious suicide attempts admitted to the emergency section or emergency rooms within 14 days of their index attempt to get treated for their physical issues.
- We will include the patients with chronic medical conditions (especially dreadful diseases), in the study, assuming that despite their constant contact with the medical facility and frequent hospital admissions due to their physical conditions, the system insensitivity could result in patients slipping through the mental health or suicidal ideation detection gateways.

Exclusion criteria for the patients include:

- We will exclude the patients with parasuicidal pauses and parasuicidal gestures to ensure the groups' homogeneity to test the intervention's effectiveness.
- Patients who do not have a consenting family member to participate will be excluded.
- We will exclude patients admitted to the psychiatric wards 14 days after the index attempt and the patients with schizophrenia spectrum disorders, severe bipolar, and severe dementia, assuming that they would require inpatient and intense care to manage their disorders, and this will prevent confounders and mixed-effects (19).
- Considering the huge treatment gap for mental disorders in India (20), the newly detected patients with serious mental disorders would be excluded from this study and will be referred to the psychiatric facility for necessary pharmacological and intense psychotherapeutic interventions.

### Ethics approval

The study received the Institutional Ethics Committee (IEC) approval on September 15, 2021: Rajagiri Institutional Ethics Committee. Ref No: RCSS/IEC/009/2021. All study participants and one of the participating family members will provide written consent to participate. Further participants will be informed about confidentiality, the voluntary nature of participation and their right to withdraw from participation at any point of study.

### Study procedure

The initial phase would focus on developing a collaborative family approach within the unique cultural milieu of Kerala, India, to maximize acceptability. The team will perform the forward/back-translation of standardized scales and indexes into the local language (Malayalam). This phase would also involve the development of an accompanying fidelity scale to guide and test the implementation of the ABFT model in the selected population so that mediators for good outcomes could be evaluated. Social Work Trainees (SWTs) supervised by the professional mental health team consisting of a psychiatrist, clinical psychologist, psychiatric social workers and psychiatric nurses would assess and provide intervention. The mental health team has supervisory, trouble-shooting, and evaluative functions. Social worker trainees would record qualitative observations regarding processes and outcomes to complement the measurement tools to enhance the overall evaluation. Focus group discussions would also be employed to review progress and address any treatment issues. The feasibility trial phase would last up to 6 months until SWTs demonstrated reasonable competence in the use of measurement tools and the intervention packages. The last phase will involve a cluster-randomized trial with a targeted sample size of 158 people who has SHB presenting at emergency departments at selected clusters in Kerala. The ABFT model will be implemented bi-weekly for 6 months, and the results will be compared with a treatment-as-usual (TAU) comparison group (medical management).

### Outcome

Study outcomes are assessed at baseline and 6-month follow-up. Demographic characteristics measured at baseline include age, gender, marital status, education, occupation, and family income. The following information on the clusters selected will be collected: number of hospital beds, patients, social workers, primary treatment modalities, and the average length of stay at the hospital. Measurement domains and time points are summarized in Table 1.

**Table 1:** Measurement domains and data collection time points.

**Variable**	**Dimensions included**	**Measurement tool**	**Data collection time points**		
			**Baseline (T0)**	**3 months (T1)**	**6 months (T2)**
Demographic assessment	Age, sex, marital status, education, occupation, and family income		✓		
Suicidality	Suicidal thoughts and ideation	Depressive Symptom Inventory–Suicidality Subscale (DSI-SS)	✓	✓	✓
Depression, anxiety, stress	Depression, anxiety, stress	DASS 21	✓	✓	✓
Social support	Emotional support, tangible support, affectionate support, positive social interaction	The MOS social support survey	✓		✓
Resilience	Resilience	The Brief Resilience Scale (BRS)	✓		✓
Customer satisfaction		Questionnaire			✓

#### Primary outcome

The primary outcome will be the total suicidality score measured by the Depressive Symptom Inventory–Suicidality Subscale (DSI-SS) (21). The DSI-SS is a 4-item self-report questionnaire designed to assess the frequency and intensity of suicidal ideation and impulses in the past 2 weeks. The scale measured suicidality on a four-point scale ranging from 0 to 3, with higher scores indicating greater severity of suicidal ideation. DSI-SS scores exhibited good internal consistency, ω = 0.90 [95% CI =0.89–0.91], convergent validity (rs = 0.52–0.74), discriminant validity (rs = 0.12–0.27) (22).

#### Secondary outcome

Secondary outcomes to be measured include depression, anxiety, mental well-being, social support, resilience, customer satisfaction, and appropriate use of primary care and community resources.

##### Depression, anxiety, stress

DASS 21 is a shortened version of Depression, Anxiety and stress scale developed by Lovibond and Lovibond (23). Participants will be asked to rate their levels of depression, anxiety and stress on a 4-point severity/frequency scales over the past week. Subscales include seven questions each which is computed to obtain total scores for depression, anxiety and stress. Higher scores on the scale indicate high severity of symptoms. The DASS 21 tool exhibits high internal consistency, with alpha coefficients of 0.85, 0.81, 0.81 respectively for depression, anxiety and stress scales and is a tested valid in Indian population (24). The study will look for a decrease in depression scores from baseline to follow-up among participants in both groups to test the effectiveness of the intervention.

##### Social support

Social support will be measured using the Medical Outcomes Study (MOS) Social Support Survey, a 19-item questionnaire measuring the availability of support in different life domains. The scale was evaluated to be reliable (all Alphas >0.91) and valid (25).

##### Resilience

The Brief Resilience Scale (BRS) measures the ability to bounce back from stressful situations in life (26). BRS is a 6-item questionnaire measuring resilience on a five-point scale ranging from one (low resilience) to five (high resilience). The scale exhibits good internal consistency with Cronbach's α = 0.71 (27).

The project team will measure customer satisfaction regarding the services at the end of the follow-up measurement using a set of questions measuring overall efficiency, professionalism, accessibility, and the overall administration of benefits.

### Intervention components

#### Intervention group: ABFT model of intervention

Participants allotted to the intervention group will receive 12 sessions of biweekly interventions spread across 6 months. Following the baseline assessment, the initial sessions will include two psycho-educative sessions for both patient and family members, focusing mainly on post-hospital engagements and crisis intervention. The introductory sessions will focus on detailed Bio-Psycho-Socio-Cultural-and-Economic assessments exploring the stressors, current coping strategies, and social support, and addressing the priority issues and crisis domains through psychological first aid. The initial sessions are followed by Attachment-Based Family Therapy involving the patients and their significant family member(s) using a structured intervention module. The intervention module will have four major components; relational reframe, developing alliance, repairing attachment, and promoting autonomy (14). Selected social work trainees from educational institutions geographically close to the selected clusters will be trained to use the module to intervene with the participants. Telephonic interventions will be provided to the recruited participants through eight different sessions focusing on various aspects. The first two sessions of telephonic intervention focused on relational reframing. The subsequent sessions are on developing therapeutic alliance work in different family contexts. The following two sessions on repairing attachment work or attachment tasks focus on improving the patient's psychosocial functioning at home and in the community. The final sessions promote autonomy by introducing more respectful and regulated interpersonal problem-solving strategies to tackle different life areas.

The last sessions of the intervention focus on facilitating the linkages with formal and informal community resources available in their locality. The intervention's various components and delivery mechanisms are detailed in Table 2.

**Table 2:** Intervention components.

**Intervention**	**Intervention components**	**Delivery**	**Human resource**
Hospital-based engagements and crisis intervention (2 sessions)	**Psycho-education Focus**-detail Assessment and psychological first aid **Session 1** • Comprehensive assessment of stressors, coping and social support, and instant emotional support through empathetic listening and validation • Psycho-education to the participants and their immediate carers about phenomenology and symptomology of suicidality **Session 2** • Addressing practical issues and use of behavioral and cognitive strategies	Direct	Social workers
Attachment-based family therapy (8 sessions)	**Relational reframe** **Focus**-Environment modification **Goals:** -Improved family communication, and using family members as change agents **Session 1:** • Identifying and articulating the unhelpful patterns in the family • Strengthening the family to address the trust ruptures, inducing a compassionate tone in conversations. **Session 2** • Revisiting current coping strategies and encouraging open and empathic conversations **Developing alliance** **Goals:** -Addressing core family conflicts and providing family members with emotional coaching the skills **Session 3:** • Identifying strengths of the clients, and Problem procedure analysis **Session 4:** • Cognitive, behavioral and emotional strategies **Repairing attachment** **Focus on clarification** **Goals:** Repairing attachment work, improving clients' psychosocial functioning, and working through attachment ruptures through attachment tasks. **Session 5:** • Direct and open communication encouraged • Working through the concerns of family members and their lives and developing trust. Emotional coaching to the family members. **Session 6:** **Strategies** • Appropriate mutual sharing is encouraged to foster mutual intimacy, mutual respect and shared responsibilities **Promoting autonomy** **Focus-insight** **Goals**-Promoting autonomy by introducing problem-solving in interpersonal situations, and engagements. **Session 7 and 8:** • Family encouraged to support the client; Social support improved • Family resources mobilized to address their unmet needs and practical requirements.	Telephonic	Social work trainees (supervised by the multidisciplinary project team)
Linkages with formal and informal Community Resources (2 sessions)	**Connecting with resources to ensure continued support** **Session 1 and 2:** **Focus**–Referral and follow up **Goals**-To facilitate the linkages with locally available formal and informal community resources Strategies: - Linking with Government welfare Departments, Non-Government, Voluntary or Philanthropic Organizations, Economic (job pool), Religious and Social resources (neighborhood groups, community, and religious organizations)	Telephonic	Social work trainees (supervised)

#### Control group: Treatment as usual

Participants recruited to the control group will receive usual care from the hospital. The usual treatment consisted of the medical management of the injuries and referral to various medical specialities and services based on their needs. Often, emphasis on the medical model makes the health system insensitive to the critical psychosocial factors associated with self-harm behavior. In Kerala, the family members or significant people accompany them and motivate them to continue the treatment. However, the option of after-treatment care will be available to patients from both groups, but they will not be assertively engaged with the intervention care package. Psychoeducation about the need for pharmacological treatment will continue in both groups whenever relevant. The family will be given additional information about the early warning signs of suicidal thoughts and ideation to their family members to detect the risk and suicidal intent. Baseline, interim, and follow-up measurements will be done using the same standardized tools for both groups.

### Human resource

The multidisciplinary team for the development and implementation of the project includes

- the lead investigators,
- medical professionals of the department of critical care and emergencies,
- professional mental health team in the hospitals and SWTs.

Social Work Trainees specializing in medical and psychiatric social work will be recruited from the educational institutions based on their readiness to volunteer for this project. The volunteered trainees will be placed in the emergency departments of the hospitals as part of their field practicum under the supervision of the mental health team and the project lead in implementing the intervention. The trainees will be encouraged to refer their participants, if necessary, to the mental health team for pharmacological and other intense therapeutic interventions.

### Statistical analysis plan

#### Sample size and power calculation

A sample size of 282 will be collected from 10 clusters, with an average of 28 required from each of the 10 clusters. The clusters receiving the FASE intervention and the clusters receiving the usual care will be randomized 1:1 ratio and 28 samples will be recruited for the study allowing for a 20 % loss in follow-up rate. The Critical z is 1.64 and *R*^2^ is 0.3 with 95% power, a two-sided alpha of 5% with a cluster effect of 0.05 results in a sample size of 256. The 95% power and additional 10% respondents would allow us to maintain at least the 80% power even after the 20% loss to follow-up. Thus, we aim to recruit 282 participants for this study.

#### Data analysis

Hard copies of data will be stored in a secure place, while soft data will be stored securely using file locks and passwords. Basic descriptive statistics will be computed for outcomes. The main effects of the interventions will be assessed on primary and secondary outcomes at different time points before and after the intervention. With the SPSS and R software, we will run the multiple regression modeling to determine the overall fit of the model and the relative contribution of each of the predictors to this variance. Multinomial Logistic Regression will be applied to study the exposure variables that are likely to contribute to the variation on the outcome variables, and discriminant analysis will be used to determine whether the clusters differ with regard to the predictors.

#### Sustainability and cost-effectiveness

Resource utilization will be recorded on all aspects of intervention implementation. Social Workers' salary, printed intervention manuals and brochures, telephone recharge expenses, train hall rent, transportation, scribbling pad and recording facility will be provided and the expenses will be documented to evaluate the cost-effectiveness of the project. Apart from 10 professional social workers employed in each cluster, no additional human resource expenses are expected as we focus on task sharing with the existing health workforce in the hospitals for implementing and supervising the intervention. The follow-up intervention by the second-year psychiatric social work students as part of their field placement reduces the human resource cost. Therefore, we will not calculate this cost within the trial. We will call or make home visits (if needed), to the participants to ensure they do not incur any additional telephone recharge costs or travel expenses. Per person cost will be calculated by total expenses divided by the number of people who received the FASE intervention. The anticipated savings from loss of life or the hospitalization costs, which the families largely incur due to repeated attempts alone, would likely offset the costs of paying social workers' salaries. If this approach proves promising in Kerala, more rigorous testing and nationwide dissemination could be possible with the support of more than 540 Schools of Social Work, most of which have field placement agreements with nearby hospitals. This family-focused model could be customized to suit the socio-cultural context of the locality. This model can be incorporated into the social work curricula through MSW internship training and post-graduate practice. Within a few years, India would have general hospital-based mental health services where virtually none currently exist. A national social work mental health organization could codify the credentialing of practice standards.

## Discussion

Self-harm behavior is one of the most common reasons for admissions to emergency departments. It is alarming to note that the risk of suicide increases 50 to 100 times within the first 12 months after an episode of SHB than the general population risk (28, 29). At least 1% of patients who present to the emergency department after self-harm behavior complete suicide within a year, with 3–5% doing so within 5–10 years (28). The sub-optimal psychosocial assessment and interventions they receive in such emergency settings are not enough to protect them from the pattern of repeated self-harm and multiple re-presentations. The reason could be ascribed to the failure to address their complex and numerous psychosocial issues, which precipitate self-harm behavior and suicide.

Socio-environmental, psychological, cultural and politico-economic factors are more relevant to studying suicide than mental disorders and biological factors in the south-east Asian region (SEAR) countries (30). Psycho-socio-environmental factors such as childhood maltreatment (31), drifting apart in relationships (32), disrupted family constellation (33), parental loss (34), discriminations and marginalization (35), and stress due to socio-politico-cultural changes in terms of collectivism to individualism (36) corroborate better with suicidality than the neurobiological correlates and biomarkers.

The tertiary prevention models of intervention programs to prevent and manage suicide are dismal in India (37), with just a few past efforts (6, 38). There is currently an alarming lack of reliable evidence regarding the psychosocial management of self-harm behavior. Furthermore, there has been very little longitudinal monitoring of this therapeutic work with suicidal people. Those management strategies that have been evaluated are inevitably medical or involve a specific psychotherapeutic intervention such as dialectical behavior therapy (11). No generally accepted care model currently exists explicitly involving short-term psychological and social interventions to reduce suicidal behavior.

Lack of consensus about what constitutes effective management strategies is a barrier to designing an appropriate care package. Psychological interventions developed in secondary care have shown some benefits. However, there are a need for such interventions to be evaluated in large populations, low and middle-income countries, especially within the setting of emergency departments, where they may need to be modified or tailored to the multidisciplinary and sensitive nature of the environment, along with the reality of time constraints.

The major component of this primary, secondary and tertiary suicide prevention model is attachment-based family therapy (ABFT), which is proven effective among adolescents (39). The primary reason to propose the ABFT model is that family members are the primary source of support for almost 90% of people with mental disorders in India. Moreover, collectivist societies like India have the luxury of resorting to family support when in need. Strong family bonds and relations can protect family members from suicidal ideation. Additionally, the family-based intervention was a practical approach in India, where family caretaking is considered a moral imperative. Therefore, the family environment modification directed toward increased support and modified faulty communication patterns has been incorporated as the model's primary focus. Evidence reiterated this assumption that enhanced family support leads to increased self-esteem, increased emotional support such as availability of family or friends for distraction in times of stress, availability of family or friends to remove means to commit suicide, and so on (40).

Although no large-scale rigorously controlled trials of family interventions have been conducted in India to date, similar approaches are promising (41–43). Teams consisting of hospital-based medical practitioners, psychiatric social workers, and psychiatric social work trainees would provide much-needed family-focused services that are relatively brief, effective, affordable, scalable, feasible, and culturally congruent (44). This intervention is not only innovative, but it is also highly significant considering the scale and importance of the problem of suicide being addressed and the potential generalizability of this model to other emergency departments throughout India.

## Conclusion

The role of a brief multidimensional intervention model focusing on family functioning and connectedness and its integration into the public health system has not been explored. Thus, further development and wide dissemination of this care package could dramatically impact post-hospitalization care of people with suicidality by helping them learn coping skills within their milieu, reducing repeated attempts. Our strategy answers the perennial call to facilitate the transfer of knowledge from clinical research into routine care that includes integrated evaluation procedures as part of the practice. The interventions focusing on enhancing follow-up care for suicide reduce the burden on the patients and their caregivers and the more extensive healthcare system. It is also hoped to develop best practices in health interventions for at-risk clients. The experience gained in conducting this study will remain with the health service and improve the service staff's skill and knowledge base to detect and manage people with self-harm behaviors.

## Ethics statement

The study is approved by the Institutional Ethics Committee of Rajagiri College of Social Sciences (Reference Number: RCSS/IEC/009/2021). The study will be conducted according to the guidelines of the Declaration of Helsinki. Participants will be recruited after obtaining consent and explaining the study.

## Author contributions

SD: conceptualization, methodology, funding acquisition, and writing—original draft. LJ: supervision and writing—review and editing. JV: conceptualization. LS, AB, and NC: writing—review and editing. All authors contributed to the article and approved the submitted version.

## Funding

This study will be funded by the Rajagiri College of Social Sciences (Autonomous). Funders have no role in the design of the study and collection, analysis, and interpretation of data and in writing the manuscript. This work is supported by Rajagiri Hospital, Aluva and Rajagiri College of Social Sciences (Autonomous).

## Conflict of interest

The authors declare that the research was conducted in the absence of any commercial or financial relationships that could be construed as a potential conflict of interest.

## Publisher's note

All claims expressed in this article are solely those of the authors and do not necessarily represent those of their affiliated organizations, or those of the publisher, the editors and the reviewers. Any product that may be evaluated in this article, or claim that may be made by its manufacturer, is not guaranteed or endorsed by the publisher.
